# Canaloplasty in the spotlight: surgical alternatives and future perspectives

**DOI:** 10.22336/rjo.2022.44

**Published:** 2022

**Authors:** Hanga Beres, Gabor Bernd Scharioth

**Affiliations:** * Aurelios Augenzentrum Recklinghausen, Germany; ** “George Emil Palade” University of Medicine, Pharmacy, Science and Technology of Târgu Mureş, Romania; *** University of Szeged, Hungary

**Keywords:** canaloplasty, glaucoma, ab interno canaloplasty, ab externo canaloplasty, Schlemm’s canal

## Abstract

The modern glaucoma surgeon is faced with many surgical alternatives for the management of glaucoma. In recent years, numerous techniques that make Schlemm’s canal (SC) more accessible for surgery by being less invasive and surgically less challenging were introduced. Since its first introduction, canaloplasty has become a well-established method of glaucoma surgery. The aim of this paper was to present an overview of canaloplasty and its modifications, and to highlight their strong points and potential drawbacks based on available data on the effectiveness of each technique. Furthermore, it offered an overview of the development of canaloplasty over time and the clinical aspects that should be considered in patient selection.

**Abbreviations:** ABiC = Canaloplasty ab interno, AH = aqueous humour, CSD = Canaloplasty with suprachoroidal drainage, IOP = intraocular pressure, MIGS = minimally invasive glaucoma surgery, OAG = open angle glaucoma, PEXG = pseudoexfoliation glaucoma, SC = Schlemm’s canal, TDM = trabeculo-Descemet’s membrane

## Introduction

There is a wide range of therapeutic options for lowering the intraocular pressure (IOP) to halt disease progression in glaucoma. Traditionally, the first line treatment is topical therapy or laser treatment, and in refractory cases, surgery is performed.

Trabeculectomy is still widely considered the gold standard of glaucoma surgery and is reported to achieve an IOP reduction between 47.73-65.48% from baseline [**1**]. This bleb-dependent surgical procedure has a high success rate [**2**], but it is also associated with severe adverse effects such as hypotony, maculopathy, phlebitis/ endophthalmitis [**3**,**4**], suprachoroidal hemorrhage and increased risk of cataract formation [**5**,**6**]. The relatively high complication rates and possible severe adverse effects have made surgeons look for other alternatives [**7**]. 

Non-penetrating approaches that target the natural outflow system, including Schlemm’s canal (SC), have long been a subject of interest. Surgical techniques aiming to reduce outflow resistance without intraocular penetration were first described in the 1960s. During sinusotomy, the external wall of SC was unroofed to facilitate aqueous humour (AH) outflow, leaving the inner wall untouched [**8**]. Deep sclerectomy was introduced in the 1980’s, which involved the excision of a deep corneo-scleral flap, leaving behind a thin trabeculo-Descemet’s membrane (TDM), and exposing both walls of the SC [**9**,**10**]. This technique was then completed with the dilation of the surgical ostia of the SC in the late 1990s (viscocanalostomy) [**11**,**12**], later surgical implants were used on the surgical site (e.g. SK Gel, T-Flux) [**13**,**14**]. The pioneers of canaloplasty were Kearney and Stegmann, Kearney introducing the circumferential viscodilation procedure to Stegmann, who then added an intracanalicular tension suture, creating the canaloplasty procedure [**15**]. By definition, canaloplasty is a non-penetrating, bleb independent procedure intending to restore the physiological outflow pathway affected in glaucoma. 

Since its first introduction, canaloplasty has proven to be safe and effective at lowering the IOP [**16**-**18**]. Canaloplasty targets the distal resistance to AH at the level of SC and collector channels in addition to its effects on the inner wall of the SC and trabecular meshwork (TM) [**19**]. When compared to trabeculectomy, canaloplasty has a better safety-profile, as it requires less intensive postoperative care and interventions, but reaches a more modest IOP reduction [**20**-**22**]. 

## Surgical technique

The first step of canaloplasty is opening the conjunctiva with either a fornix or limbus-based incision. Next, a rectangular shaped superficial scleral flap of about one-third of the scleral thickness is created. Even though bleeding is anticipated, diathermy of episcleral vessels should be avoided to preserve outflow [**23**]. Then, a slightly smaller and deeper scleral flap is sculpted just above the choroidal plane and extended towards the scleral spur. A clean dissection of the SC is performed. To minimize the risk of perforation of the TDM, a paracentesis should be performed no later than this point. Afterwards, a trabeculo-Descemetic window is created, followed by the removal of the deep sclero-corneal flap.

**Fig. 1:**
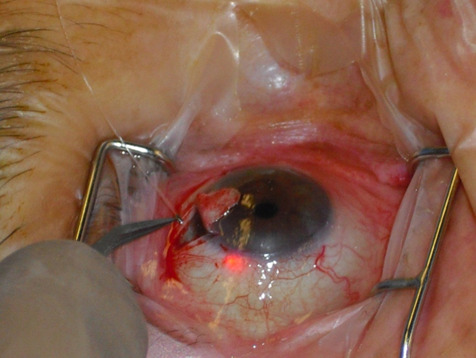
iTrack advancing through the SC

**Fig. 2:**
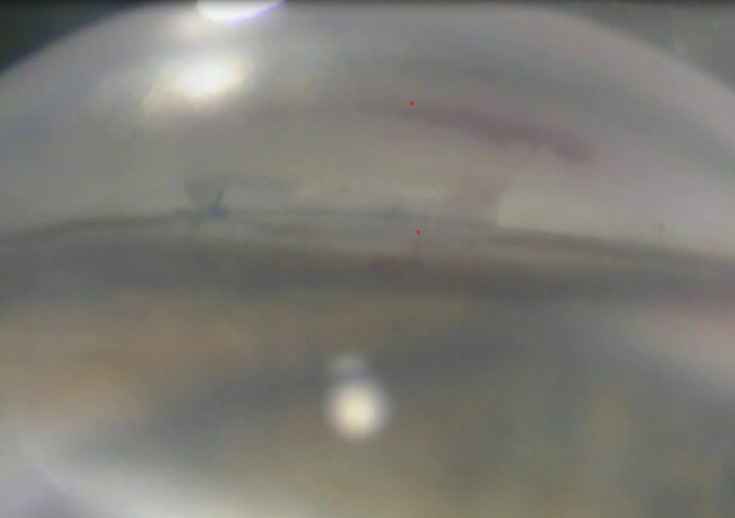
Intraoperative gonioscopic view of the scleral lake and descemetic window. Tensioning suture and knot in place, heavily pigmented TM outside of the deep sclerectomy where it was peeled off

The ostia of SC are viscodilated using a high viscosity OVD with a microcannula. Then, a microcatheter (iTrack 250, Ellex iScience, Inc., Freemont, CA, USA) is inserted into one of the surgically created ostia and advanced throughout the whole circumference of the SC (**Fig. 1**). When the distal tip of the catheter is exposed, a 10/ 0 polypropylene suture is tied to the distal tip and the microcatheter is withdrawn pulling the suture into the canal. In this phase of the surgery, it is recommended to use a high weight OVD to enhance the postoperative outcome. The OVD breaks adhesions within the SC, stretches the trabecular plates, thus facilitating AH outflow into SC. It also separates herniations of the inner wall of the TM into the outer collector channels [**24**,**25**]. After removing the microcatheter, the two ends of the suture are knotted together, placing tension on the tissue, thus distending the TM inwards. At this point, if possible, the inner wall of the SC with the endothelium and juxtacanalicular meshwork may be peeled to facilitate outflow (**Fig. 2**) [**23**]. Finally, the superficial scleral flap is tightly closed with 5 to 7 Vicryl or nylon sutures and OVD is injected under the scleral flap to minimize bleb formation and prevent scarring [**26**]. The anterior chamber is refilled with balanced salt solution to a normal to slightly elevated IOP. The conjunctiva is also repositioned and sutured in a watertight fashion.

## Patient selection

Canaloplasty is usually indicated in mild-to-moderate open angle glaucoma (OAG) cases, but is also suitable for angle closure glaucoma in combination with cataract extraction [**27**]. This procedure is also effective in cases of pigmentary glaucoma [**28**] or pseudoexfoliative glaucoma (PEXG) [**29**,**30**]. Canaloplasty with 360-degree trabeculotomy is also a good option for congenital glaucoma and juvenile glaucoma [**31**,**32**]. It may also be considered in some cases of secondary glaucoma such as uveitic glaucoma [**33**] and in corticosteroid-induced glaucomas [**34**]. Both canaloplasty and ab externo trabeculotomy were reported to be a safe and efficient IOP lowering alternative after failed trabeculectomy with both intact and disrupted SC [**35**-**37**]. Furthermore, canaloplasty can be performed in combination with phacoemulsification, which causes an additional slight reduction in the IOP on long term [**38**]. Finally, canaloplasty might be a better option for young patients with moderately elevated IOP, contact lens wearers, and patients with intolerance to topical therapy. 

Contraindications for canaloplasty include angle-closure glaucoma and narrow-angle glaucoma (if the patient does not undergo concurrent lens extraction), secondary glaucoma (neovascular glaucoma, and posttraumatic glaucoma with angle recession) without the previously mentioned exceptions, and in cases with underlying damage to SC due to previous ocular surgery or extensive thermal laser trabeculoplasty with peripheral anterior synechiae [**24**]. 

## Modifications to canaloplasty

Over the years, several surgical techniques were introduced, aiming to make canaloplasty less invasive, surgically less challenging and more accessible.

**Table 1:** Canaloplasty and modifications to the technique

Canaloplasty features	Classic Canaloplasty	Cathetherless Canaloplasty	Canaloplasty with Glaucolight	Canaloplasty with Suprachoroidal Drainage	Mini-canaloplasty	Ab interno canaloplasty (ABiC)
Advantages	The most data available	The most affordable	Lower risk of Descemet membrane detachment	Possibly stronger IOP reduction, easier localization of SC	Shorter surgery times	Minimally invasive, sclera and conjunctiva remain intact
Disadvantages	Longer operating time	Higher risk of creating false passages	Commercially not available	Risk of choroidal lesion	Possibly a more modest IOP reduction	Surgically challenging, gonioscopic view, higher costs
Approach to Schlemm’s canal	Ab externo	Ab externo	Ab externo	Ab externo	Ab externo	Ab interno
Catheter type	iTrack	Prolene 6x0	Glaucolight	iTrack	iTrack	iTrack/ Omni System/Visco360
Superficial flap	5x5mm	5x5mm	5x5mm	4x4,5mm	4x1mm	no
Deep scleral flap	4,5x4mm	4,5x4mm	4,5x4mm	3,5x4mm	1x1	no
Viscodilation	yes	no	no	yes	yes	yes
Tensioning suture	yes	yes	yes	yes	yes	no
Intrascleral lake	yes	yes	yes	yes	no	no
Suturing of the conjunctiva	yes	yes	yes	yes	no	no

## Cathetherless canaloplasty

**Fig. 3:**
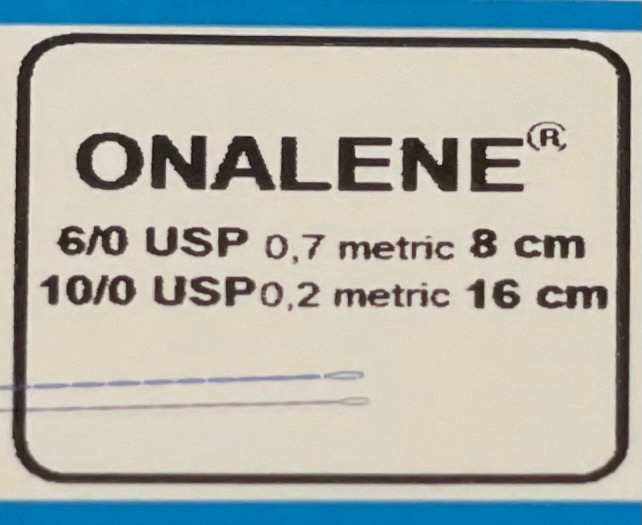
6/ 0 and 10/ 0 double helix suture, Onalene® (Geuder, Germany) used for sutureless canaloplasty

As the illumination equipment is expensive, surgeons tried different alternatives to replace the microcatheter. Beck first used a 6/ 0 polypropylene suture in circumferential cannulation in 360-degree trabeculotomy for primary congenital glaucoma in 1995 [**39**]. Later, this technique was described for the treatment of OAG patients [**40**,**41**]. After the preparation of the deep scleral flap, the ostia of SC are enlarged by using an OVD and a 6/ 0 polypropylene suture is introduced into the canal. The suture probe is blunt and slightly curved or formed in a double spiral. After circumferential catheterization, the distal tip is exposed at the other ostium and a 10/ 0 prolene suture is fastened to it. On removal, the 10/ 0 prolene suture remains in the canal secured through a slip knot or a four-throw knot. In recent prospective studies [**42**-**44**], a twisted 6/ 0 polypropylene suture was used for the cannulation of the SC (**Fig. 3**). The authors reported that the smooth tip of the loop ensures an atraumatic probing of the SC, while the double helix configuration provides a good rigidity to the suture. According to the authors, the success rate of circumferential cannulation by twisted 6/ 0 sutures is up to 90%, which is not inferior to the previously reported cannulation rates in conventional canaloplasty [**16**,**45**] or by the Visco360 and Omni System [**46**].

## Canaloplasty with Glaucolight (D.O.R.C. Dutch Ophthalmic Research Center, Zuidland, the Netherlands)

Glaucolight was proposed by Scharioth, offering a more affordable alternative to the iTrack microcatheter [**41**,**47**]. The probe is a specially designed of lightfiber with an atraumatic tip. Having a smaller diameter (150 µm/ 40G), it allows more flexibility while passing through SC. With its integrated, sterile and battery-powered LED light source, Glaucolight offers good visualization during cannulation without the need to connect it to an external light source. A major difference between the two microcatheters is that iTrack allows for a 360-degree injection of OVD. When using Glaucolight, only the ostia of the SC are viscodilated to facilitate the cannulation, without circumferential viscocanaloplasty. Similar to standard canaloplasty, after 360-degree cannulation, the distal tip is tied to a 10/ 0 polypropylene suture that is then pulled into SC. Next, a loop is created by tying the ends of the suture together. Finally, a deep sclerectomy is performed and the superficial flap is closed in a water-tight manner.

Vastarbis et al. compared the efficacy of canaloplasty carried out with iTrack and Glaucolight in a retrospective study [**48**]. In the 12-month post-op follow up, the two surgical procedures showed similar IOP lowering effects. The authors also underlined that the risk of Descemet membrane detachment is lower with Glaucolight assisted canaloplasty. Scharioth also reported a lower number of Descemet membrane detachment with Glaucolight [**47**]. The authors argue that this complication is more frequent while using the iTrack because of the circumferential viscodilation [**47**,**48**].

## Canaloplasty with suprachoroidal drainage (CSD)

This surgical technique was first introduced by Szurman in 2016. The main difference is the dissection of the deep scleral flap. In contrast to traditional canaloplasty, the second flap is not dissected in a lamellar fashion, but is excised in a full thickness block to the choroid, exposing the suprachoroidal space. After exposing SC, it can either be cannulated with a microcatheter (e.g. iTrack) or with a prolene suture, as previously described. Finally, the deep scleral flap is excised and the superficial flap is closed in a water-tight manner. The authors argue that an additional IOP lowering effect can be achieved in CSD because it also alters the uveoscleral aqueous outflow, thereby allowing AH to drain directly into the suprachoroidal space [**49**].

In 2016, Seuthe et al. published a retrospective study on many patients (n=417) comparing conventional canaloplasty (n=180 eyes) to CSD (n=237 eyes). The mean IOP reduction was significantly higher following CSD than after standard canaloplasty (35.9% vs. 31.2%; from baseline 20.9 ± 3.5 mmHg to 13.1 ± 2.5 mmHg vs. from baseline 20.8 ± 3.6 mmHg to 14.0 ± 2.6 mmHg). The number of IOP-lowering medications decreased after CSD from 3.5 ± 0.9 to 0.7 ± 1.0, and after conventional canaloplasty from 3.4 ± 0.9 to 0.8 ± 0.9, with more patients being medication-free at a 1-year follow up (56.9% vs. 45.4%) [**50**]. Seuthe et al. also compared the efficacy of CSD as a standalone procedure and combined with phacoemulsification in a retrospective setting. CSD achieved an IOP reduction of 37% (from 20.9 ± 3.6 mmHg to 13.2 ± 2.6 mmHg, n=193 patients), whereas CSD combined with phacoemulsification reached a significantly higher IOP reduction of 47.4% (from 23.2 ± 5.1 mmHg to 12.2 ± 1.7 mmHg, n=135 patients) [**51**]. The efficacy of CSD was also evaluated in patients with PEXG. The authors reported an IOP reduction of 45.8% after 12 months (from baseline 23.4 ± 5.1 mmHg to 12.7 ± 2.2 mmHg) and 45.1% after four years (12.8 ± 2.2 mmHg) and a significant decrease in IOP-medication (from 3.4 at baseline to 0.6 after 12 months and to 1.0 after four years) [**52**]. None of these studies reported any serious, sight-threatening complications.

## Mini-canaloplasty

This method was introduced by Rękas et al. and involves a mini-incision technique for accessing the Schlemm’s canal without the need to prepare the classical TDM and having to close sutures. The main difference of this variation is the size of the flaps created, the superficial flap having a diameter of 4.0 x 1.5 mm, and the deep flap 1.0 x 1.0 mm. After locating Schlemm’s canal, the ostium can be probed with iTrack and an OVD is injected at every 2 hours on removal. The tensioning suture is placed in the same way as in conventional canaloplasty. Finally, without the removal of the deep scleral flap or dissection of the intrascleral lake or the TDM, the conjunctiva is closed with diathermy. As there is no intrascleral lake, the hypotensive effect will only be induced thorough the tensioning suture in the Schlemm’s canal. The authors’ preliminary results show an IOP reduction of 23% (from baseline 18.0 ± 8 mmHg to 15.5 ± 4.1 mmHg) and a significant reduction in the number of medications (from baseline 3 ± 1 to 0.25 ± 1.0) at the end of their observation period [**53**]. 

## Canaloplasty ab interno (ABiC)

This modified technique classifies as minimally invasive glaucoma surgery (MIGS) because it involves the viscodilation of Schlemm’s canal via ab-interno approach using a clear corneal incision. The viscoelastic primed iTrack microcatheter is introduced through a side port incision at the 12 o’clock position and directed towards the nasal iridocorneal angle. A 1.8 mm clear corneal incision is created temporally and, under gonioscopic view, a small goniotomy is created. The iTrack microcatheter is then introduced into Schlemm’s canal and threaded 360 degrees. Viscoelastic is injected during removal. No tensioning suture is placed in this method [**54**].

In their retrospective study, Gallardo et al. compared the efficacy of ABiC as a stand-alone procedure (n=41 eyes) to ABiC combined with phacoemulsification (n=34 eyes). They found no statistically significant difference in the reduction of the IOP levels (32.8% ABiC vs. 31.7% ABiC combined with phacoemulsification), nor in the number of IOP-lowering medications needed (36% vs. 40% of the patients were medication free) at the end of the 12-month follow-up period [**54**]. Gallardo et al. also compared the efficacy of ABiC and standard canaloplasty in a retrospective case series, finding no statistically significant IOP reduction between the two groups, however the number of participants were very limited (n=12) [**55**].

An interesting development for ABiC is the OMNI Surgical System device (Sight Sciences, Menlo Park, CA), allowing two surgical procedures at once: goniotomy and canaloplasty. After engaging the tip into the TM, an opening into SC is created. The microcatheter is then navigated through SC for 180 degrees in each direction. As it is retracted, viscoelastic is released from the tip of the catheter to viscodilate SC. Hughes and Traynor reported a mean IOP reduction of 36% (from baseline 24.5 ± 8 to 15.8 ± 2.5) and a 32% reduction of glaucoma medications (from baseline 2.5 ± 1.3 to 1.7 ± 1.5) when performing ABiC with the Visco360 or the OMNI Surgical System [**46**]. Other authors also reported similar results with ABiC as a standalone procedure or in combination with phacoemulsification [**56**-**58**].

Since the first introduction of the ab-interno approach in 2018 [**54**], numerous studies were carried out using the OMNI Surgical System. Just recently, the 12-month results of a large, multicenter, prospective study were published (GEMINI Study). The patients underwent 360-degree canaloplasty and 180-degree trabeculotomy using the OMNI Surgical System in combination with phacoemulsification. The authors report that 84.2% of the eyes achieved IOP reductions greater than 20% from baseline and 80% of eyes were medication-free at 12-month follow-up [**59**]. In the ROMEO Study, patients underwent the same procedure as in the GEMINI Study. This large, multicenter, retrospective study also came to similar results in terms of IOP reduction (79% of eyes had a 20% IOP reduction or had an IOP between 6 and 18 mmHg), but only 33% were medication free on the 12-month follow-up [**60**].

## Discussion/ Future perspectives

In the last decades, the management of glaucoma has undergone major changes. New surgical techniques, like canaloplasty, aim at minimizing ocular trauma whilst delivering good IOP lowering results.

One advantage of leaving a tensioning suture in SC is the possibility to perform a 360-degree suture trabeculotomy, a minimally invasive, low risk revision surgery, if IOP reduction is insufficient. Under gonioscopic view, end-gripping forceps are introduced into the anterior chamber to grasp and remove the suture through the TM. Seuthe et al. reported a 41.2% of IOP reduction (from 22.8 ± 6.7 mmHg to 13.4 ± 2.3 mmHg) and a decrease in medication need (from 2.7 ± 1.4 to 1.6 ± 1.2) at the 12-month follow-up [**61**].

Grieshaber et al. also described a modified dissection technique, called flap sparing canaloplasty, which does not involve the creation of an intrascleral lake. To access Schlemm’s canal after a limbal peritomy of the conjunctiva, a radial vertical cut-down incision is created over the limbus until the Schlemm’s canal is opened. After viscodilation with a microcannula, they implanted Stegmann Canal Expanders, then the sclera is closed with a watertight fashion. The authors report a 48% reduction of IOP (from baseline 31.9 mmHg ± 6.0 to 15.2 mmHg ± 1.95) at the 12-month follow up [**62**].

In conclusion, canaloplasty is a good option in mild to moderate OAG cases, reaching an approximately 40% IOP reduction. There is a lot to consider when choosing the appropriate modification of canaloplasty, such as surgical experience, affordability, availability of surgical instrumentation and available data that support surgical success. Although there are a lot of studies that show short-term success, there are less studies available presenting mid-term results [**17**,**45**,**63**,**64**] and almost no data is available on long-term outcomes. Further studies are needed to offer a better understanding of these procedures and to determine if ab-externo techniques achieve a better IOP reduction.

**Conflict of Interest statement**

The authors state no conflict of interest.

**Acknowledgements**

None.

**Sources of Funding**

None.

**Disclosures**

None.
